# Spontaneous muscle haematomas: management of 10 cases

**Published:** 2014-04-08

**Authors:** V Palatucci, G Lombardi, L Lombardi, F Giglio, F Giordano, D Lombardi

**Affiliations:** 1 Department of General and Emergency Surgery, University of Salerno, Italy; 2 Operating Unit of Emergency Surgery, “S. Maria della Pietà” Hospital of Nola, Napoli, Italy; 3 Division of Anesthesiology and Intensive Care Unit, “S. Maria delle Grazie” Hospital of Pozzuoli, Napoli, Italy; 4 Department of Radiology, Federico II University of Naples, Italy

**Keywords:** muscle haematoma, anticoagulant therapy, rectus sheath, gluteal muscle

## Abstract

This is a retrospective study about the treatment of spontaneous muscle haematomas (SMH) that are an uncommon disease that occurs especially in elderly patients with acquired coagulopathy.

We report the management of 10 cases admitted to our Emergency Surgical Unit (ESU) between March 2011 and October 2012.

For this analysis we have considered some parameters such as age, drug history, current symptoms, location of the haematoma, cause, and imaging examination. Our attention focused on: clinical presentation, differential diagnosis, diagnostic imaging techniques and treatments.

## I. INTRODUCTION

Spontaneous muscle haematoma (SMH) is an uncommon condition often overlooked or misdiagnosed (1), and potentially life-threatening, particularly in frail and elderly patients. For a quick diagnosis and to choose the best treatment, a high degree of suspicion is necessary especially in patients with acquired coagulopathy (e.g., oral anticoagulant therapy) .

Most times, muscle haematomas affect the abdominal rectus sheath or the gluteal muscles.

Several predisposing or contributing factors were described (2, 3); the most frequent are: minor trauma, increased abdominal pressure (sneezing, straining, coughing, gagging), anticoagulation medications, hypertension and iatrogenic causes (e.g., intramuscular injections or laparoscopic surgery).

We report our experience in the management of 10 cases diagnosed with SMH.

## II. METHODS

We have examined retrospectively the medical history, the treatment and the outcome of patients admitted to our Emergency Surgical Unit (ESU) with a diagnosis of SMH.

The following parameters were taken into consideration: age, sex, drug history, past medical history, current symptoms, location of the haematoma, causes, haemoglobin levels and INR on admission, imaging examinations in Emergency Room (ER), treatment, days of hospitalization, outcome of the disease (table 1).

The imaging examinations (US, CT or both) were carried out according to the diagnostic suspicion of the emergency staff, to the clinical presentation of pain and to comorbidities.

The treatment could be conservative or surgical depending on the haemoglobin values and on the diagnostic imaging signs of active bleeding.

When imaging examinations didn’t show signs of active bleeding, but the haemoglobin values were inferior to 7,5 g/dl (or superior to 7,5 g/dl but quickly dropped in patients with ischemic heart disease) a blood transfusion was done. In some of the cases with acquired coagulopathy, conservative treatment also consisted of analgesia and recoagulation therapy (fresh frozen plasma, vitamin K, protamine sulphate). If the haematoma was small, did not increase in volume (no active bleeding in follow-up imaging examination),and the patient was hemodynamically stationary (blood pressure, cardiac frequency, haemoglobin were constant),surgical evacuation was avoided. The monitoring of haemoglobin values and haematoma volume by US or TC are imperative: an increase in volume of haematoma associated with a decrease of haemoglobin are clear indication for surgical intervention.

## III. RESULTS

Ten patients were admitted to the ESU at our Institution (4 males and 6 females) between March 2011 and October 2012.

The main clinical features of these patients are summarized in Table 1.

The mean age was 67.6 (range: 39–84) with a predominance of females (60%) over males (40%).

Eight patients were receiving anticoagulant / anti-platelets therapy for different underlying disease:

-mechanical aortic valve (10%)
-ischemic heart disease (20%)
-pulmonary thromboembolism (10%)
-hip fracture (10%)
-stroke (20%)
-deep vein thrombosis (10%)

INR values ranged from 1.08 and 3.11 (Mean value 2.079). All patients were anemic with Hb ranging from 6.8 g/dL and 10.9 g/dL

All patients complained acute pain at the site of onset of the haematoma.

Diagnosis were obtained in the Emergency Room: in 1 patient by only using the ultrasound scan (US), in 3 patients by using only a Computerized Tomography (CT), and in 4 patients with both imaging techniques (US and CT).

In two cases the diagnosis was easily made on the basis of the clinical examination and no imaging was done: in these cases there was a large ecchymosis on the skin of the gluteus and a hard mass was palpated.

As for these ten patients, 4 were treated surgically with incision and drainage of the haematoma; 4 patients were treated with conservative therapy (blood transfusion and re-coagulant therapy, if applicable); whereas 2 patients underwent surgical excision and drainage as well as receiving blood transfusion.

In six cases of haemodynamic instability, we preferred “surgical evacuation of haematoma and the ligation of the vessel” to “angiography and embolization of bleeding vessel” due to the risk of muscle ischemia. We haven’t taken into consideration the interventional radiology procedures we have been using in our short experience, but we think that they should be considered if there are contraindications to surgery (difficult surgical access site, serious health conditions, contraindication to anaesthesia)and if surgery fails and a second bleeding offence occurs.

One patient died of septic shock due to the infection of the haematoma related to immune depression. The remaining patients were discharged home with a mean length of hospital stay of 7,5 days (range: 3–17) influenced by the comorbidities.

## IV. DISCUSSION

The real incidence of SMH is still unknown because often this condition is misdiagnosed or overlooked. Titone et al. (4) reported the most common diagnostic errors of SMH that include: strangulated hernia (12%), twisted ovarian cyst (10%), intestinal obstruction (8%), abdominal neoplasm (4%), perforated colon (4%), appendicitis (4%). The differential diagnosis must be made with the causes of pain all in the right or left iliac fossa in case the haematoma is localized in the abdominal wall (5), or with the causes of spinal root compression if the regions affected are the gluteal or lumbar ones.

However, the most frequent location of the SMH, as also confirmed by our observation, is the rectus abdominal muscle sheath.

Bleeding is caused by injury in the upper or lower epigastric arteries or by direct damage to the rectus abdominal muscles (6).

At the site of the lesion, in addition to the pain reported by the patient, a non-pulsatile mass can be observed, often with ill-defined edges that may or not may be accompanied by ecchymosis.

A high index of suspicion for SMH should be maintained in patients with a history of anticoagulant therapy. These drugs are likely to be the single most important risk factor In our small group 8 out of 10 patients were under treatment with an anticoagulant therapy.

A reduced blood level of haemoglobin and abnormalities in the coagulation profile should also raise the index of suspicion of SMH and prompt further work up. Diagnostic imaging techniques such as ultrasonography (US) and/or computerized tomography (CT) are usually used to obtain diagnosis.

**Fig 1.**
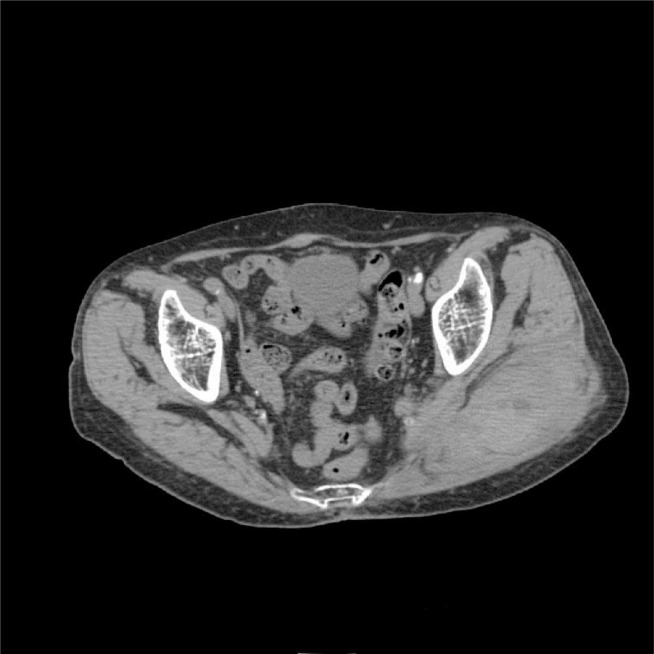
Haematoma between left gluteal muscles

**Fig 2.**
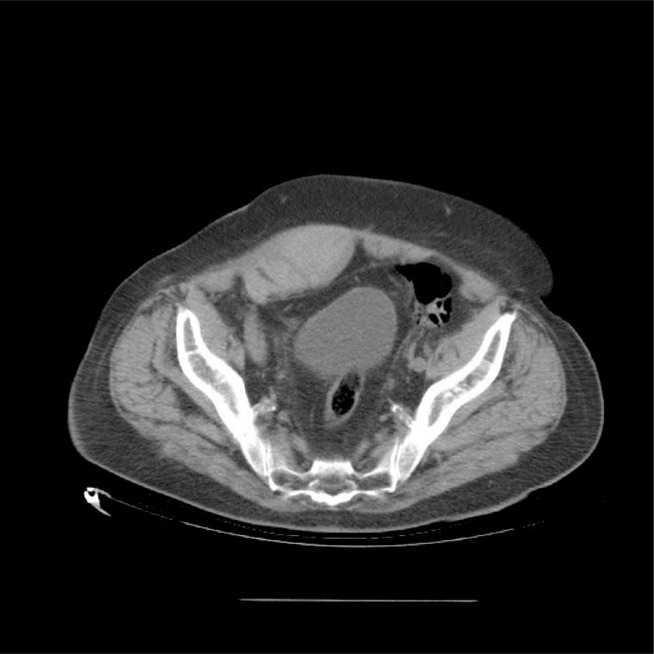
Right rectus sheath haematoma

Computerized tomography has a higher diagnostic yield than US, because it has higher sensitivity and specificity (8). In two cases reported by Siddiqui et al. (9) two muscle haematomas seen on CT were not apparent on ultrasound. On contrast-enhanced CT, muscle haematomas appear as hyperdense lesions (Fig. 1) (Fig.2), their margins are regular when they are delimited by the muscle sheath or the edge of the muscle itself. A post-enhancement hyperdense area at the core of the lesion is a sign of active bleeding.

If the haematoma interests the rectus abdominal muscles, the presence of extravasation in the abdominal cavity has to be excluded. US scan has a lower diagnostic yield, but is very useful to monitor the size of a haematoma, due to its repeatability and low invasiveness.

In a recent study on 78 patients, the extension of rectus sheath haematoma below the “arcuate line” and the number of blood transfusions required has been correlated to a potential higher risk for hemodynamic instability and mortality. Although no statistically significant prognostic risk factors of haemodynamic instability could be identified, the authors believe that a large haematoma can be predictive of haemodynamic instability.

Furthermore, repeated blood transfusions could cause consumption coagulopathy leading to death. Therefore, in these cases repeated CT scanning should be mandatory (15).

MRI can be used in long-standing haematomas for the differential diagnosis of a soft tissue tumour and a haematoma when CT scan is not conclusive (10).

Conservative treatment consists of analgesia and recoagulation therapy.

If the patient has a clotting disorder it may be necessary to give vitamin K, fresh frozen plasma, recombinant clotting factors or protamine sulphate (in patient under treatment with heparin) (6).

The need for a blood transfusion has to be gauged on the hemodynamic status and comorbidities of the patient (e.g., ischemic heart disease).

An active bleeding that does not tend to stop spontaneously can be treated either by interventional radiology procedures (angiography and embolization of the bleeding vessel) (11,12), or by surgical evacuation of the haematoma and the ligation of the vessel.

After two embolization procedures on the same patient, however, there is a high risk of muscle ischemia and surgery becomes mandatory (13). Also haemodynamic instability and suspicion of sepsis are an absolute indication to surgery (15). Surgery can be complicated by the difficulty in identifying the bleeding vessel in the context of the haematoma (13). Occasionally, haemostasis can be achieved by removing the haematoma and packing the cavity.

Reportedly, the mortality of SMH ranges from 4% to 20% (14) and includes patients treated conservatively as well as those who undergo surgery. This can be due to hemodynamic instability or to the presence of comorbidities. Furthermore, infection of a muscle haematoma can lead to sepsis, SIRS and septic shock.

In the presence of risk factors, SMH must be kept in mind as the differential diagnosis in cases of pain of lower abdominal quadrants, as well as, lumbar or gluteal pain, to avoid incorrect and unnecessary surgical procedures and to minimize mortality and morbidity in this group of patients.

Our experience suggests that therapeutic options depend on the hemodynamic stability of patient and his comorbidities. Surgery is mandatory if bleeding does not tend to stop spontaneously or with medical treatment and if signs of haematoma infection are present. Interventional radiology procedures are a valid alternative to surgery but with the risk of muscle ischemia. Due to rare occurrence of this condition and the lack of experience gained the subject until it’s necessary to store future cases of spontaneous haematomas in order to determine new therapeutic and diagnostic strategies and new treatment protocols .

## Figures and Tables

**TABLE 1.** TABLE WITH PARAMETERS CONSIDERED IN OUR STUDY

	**Sex**	**Age**	**Previous medication (underlying disease)**	**Symptoms/location of the haematoma**	**Cause**	**Hb on admission (g/dL)**	**INR on admission**	**Imaging in ER**	**Treatment**	**Hospital stay (days)**	**Outcome**
**Pt 1**	F	84	Warfarin + Anti-platelets therapy (mechanical aortic valve)	low back pain/left gluteus	Intramuscular injections	10.4	2.41	CT	incision and drainage	12	death
**Pt 2**	M	76	Warfarin (ischemic heart disease)	Abdominal pain/ Right rectus sheath	Indeterminate	7.1	2.71	US + CT	blood transfusion	6	discharged
**Pt 3**	M	70	Warfarin (pulmonary embolism)	low back pain/ left gluteus	Intramuscular injections	6.8	3.11	none	blood transfusion + incision and drainage	14	discharged
**Pt 4**	F	61	low molecular weight heparin (hip fracture)	Abdominal pain/ Right rectus sheath	Minor trauma	8.4	1.08	US + CT	incision and drainage + blood transfusion	17	discharged
**Pt 5**	F	39	none	low back pain/right gluteus	Intramuscular injections	8.6	1.38	none	incision and drainage	3	discharged
**Pt 6**	M	69	Warfarin (ischemic heart disease)	Abdominal pain/ Right rectus sheath	Indeterminate	7.3	2.54	CT	blood transfusion	7	discharged
**Pt 7**	F	58	Anti-platelets therapy (stroke)	Abdominal pain/ Right rectus sheath	Indeterminate	7.5	2.01	CT	blood transfusion	5	discharged
**Pt 8**	F	80	Anti-platelets therapy (stroke)	Abdominal pain/ left rectus sheath	Indeterminate	7	1.99	US	blood transfusion	5	discharged
**Pt 9**	F	68	none	Abdominal pain/ left rectus sheath	Indeterminate	10.5	1.56	US + CT	incision and drainage	7	discharged
**Pt 10**	M	71	low molecular weight heparin (deep vein thrombosis)	Abdominal pain/ Right rectus sheath	Indeterminate	10.9	2.00	US + CT	incision and drainage	11	discharged

**Abbreviations**. PT=Patient; HB=Haemoglobin; ER= Emergency Room
